# StarTRAC-*CYP2D6*: a method for *CYP2D6* allele-specific copy number determination using digital PCR

**DOI:** 10.3389/fphar.2025.1715830

**Published:** 2025-12-02

**Authors:** Wendy Y. Wang, Erin C. Boone, Lancy Lin, David Joun, Andrea Gaedigk

**Affiliations:** 1 Division of Clinical Pharmacology, Toxicology and Therapeutic Innovation, Children’s Mercy Research Institute (CMRI), Kansas City, MO, United States; 2 Genetic Sciences Division, Thermo Fisher Scientific, Waltham, CA, United States; 3 School of Medicine, University of Missouri-Kansas City, Kansas City, MO, United States

**Keywords:** CYP2D6, copy number variation, allele-specific PCR, digital PCR, genotyping, Absolute Q, DNA concentration

## Abstract

**Background:**

Accurate *CYP2D6* genotyping is essential for pharmacogenetic-guided prescribing of many clinically used medications. While digital PCR is increasingly used to determine copy number (CN) status for accurate phenotype prediction, there are few convenient methods to discriminate duplicated alleles and diplotypes such as CYP2D6*2/*4x2 and *2x2/*4, which predict intermediate and normal metabolizer phenotypes, respectively. We developed a novel method, StarTRAC-*CYP2D6*, “**T**argeted **R**eporting of **A**llele-specific **C**NV”, for determining the number of specific *CYP2D6* star allele copies. Additionally, we explored the impact of DNA input and interfering variants on CN assay performance.

**Methods:**

Coriell samples (n = 17) representing various *CYP2D6* star alleles and structural variants were tested on the QuantStudio™ Absolute Q™ Digital PCR System using the previously established research use only one-pot workflow and seven multiplexed TaqMan™ genotyping assays. Each multiplex targets a core variant found in commonly observed gene duplications: g.100C>T, g.1022C>T, g.1847G>A, g.2851C>T, g.2989G>A, g.3184G>A, and g.4181C. The multiplex contains targeted genotyping probes labeled with FAM, VIC and JUN, and either RNaseP or *TERT* labeled with ABY, serving as 2-copy gene references.

**Results:**

The CN of the specific alleles represented in this study include: CYP2D6*1, *2, *4, *10, *17, *29, *41, *45, *36, *68, *154, and *158. Allele-specific CN ranging from 0 to 4 copies were reliably resolved. All samples with duplications/multiplications were accurately discriminated with their expected star allele. For CN up to 6 copies, a total DNA input of 200 ng maintained CN integrity and variants interfering with primer/probe binding had a range of effects on CN determination.

**Conclusion:**

StarTRAC-*CYP2D6* is an effective, stand-alone method for determining the CN of specific star alleles. DNA quantity and possible interfering variants should be considered when optimizing workflows and accurately interpreting results. Protocols are currently being developed for higher-plex CN testing and discrimination.

## Introduction

Pharmacogenetic (PGx) testing for *CYP2D6* is increasingly used to predict a patient’s metabolizer status to inform individualized drug therapy. Over 20% of clinically prescribed medications (Saravanakumar et al., 2019) are metabolized by this important pharmacogene (Jarvis et al., 2019; Nofziger et al., 2020; Taylor et al., 2020). Additionally, Clinical Pharmacogenetic Implementation Consortium (CPIC) has published several guidelines for *CYP2D6* gene-drugs pairings that are used across multiple disciplines such as behavioral/mental health (Hicks et al., 2017; Brown et al., 2019; Bousman et al., 2023), pain (Crews et al., 2021), cardiology (Duarte et al., 2024), and oncology (Bell et al., 2017; Goetz et al., 2018). Given its clinical importance, the Association for Molecular Pathology (AMP) has published recommendations for clinical *CYP2D6* star allele testing in conjunction with these guidelines. Noteworthy, testing for gene copy number variation (CNVs) is included in both Tier-1 and Tier-2 recommendations (Pratt et al., 2021).

The Pharmacogene Variation Consortium (PharmVar) serves as a repository and provides standardized star (*) allele nomenclature for *CYP2D6* and other important pharmacogenes (Gaedigk et al., 2018; 2021). There are currently over 175 star alleles for *CYP2D6,* many of which can occur in duplication (*x2*) or multiplication (*xN*) arrangements. CNVs also include gene deletions (CYP2D6*5) and hybrid gene copies, which contain parts of the *CYP2D7* pseudogene (CYP2D6*13, *36, *68, among others). In addition, haplotypes can include complex structural variants (SVs) consisting of two or more nonidentical gene copies that may also include hybrids. An in-depth summary of *CYP2D6* SVs can be found on the PharmVar *CYP2D6* page (https://www.pharmvar.org/gene/CYP2D6; last accessed 29 September 2025) and the PharmVar Tutorial on *CYP2D6* Structural Variation Testing and Recommendations on Reporting (Turner et al., 2023). The sheer extent and complexity of SVs add an extra layer of challenges to accurately determine *CYP2D6* genotype (Nofziger and Paulmichl, 2018; Turner et al., 2023). With the frequency of SVs in the United States being estimated at 3.5%–8.4% (depending on ancestry), it is imperative for any *CYP2D6* genotyping platform or implementation program to accurately capture SVs (Black et al., 2012; Del Tredici et al., 2018; Patel et al., 2024).

Current stand-alone CNV methodologies include qualitative testing using long-range PCR (XL-PCR) and quantitative testing of gene regions using qPCR and digital PCR (dPCR) approaches. Other platforms include capabilities for simultaneous CNV detection and genotyping but may only be economical in high-throughput conditions; some of these platforms include the Agena MassArray, the Illumina Infinium Global Diversity Array with Enhanced PGx, and the Thermo Fisher Axiom PangenomiX Array. Next-generation sequencing methods such as long-read whole genome sequencing and single-molecule sequencing, *i.e.*, PacBio HiFi long-read and Nanopore sequencing, can also inform copy number status (Turner et al., 2023). However, these platforms may not be ideal for low-to medium-throughput laboratories because of the expense, requirements for higher sample throughput for cost viability and/or labor constraints. Depending on the method, they can also be bioinformatically intensive for routine PGx testing compared to array-based methods.

We have previously established a robust digital PCR method to simplify CNV testing by implementing a one-pot restriction enzyme digestion workflow and multiplexing three *CYP2D6* target regions (5′UTR, intron 6, and exon 9) using the Applied Biosystems™ QuantStudio™ Absolute Q™ dPCR System (Wang et al., 2024). However, only resolving the copy number of specific gene regions without discriminating duplicated alleles may be insufficient for informing clinical action. For example, a CYP2D6*2/*4 genotype with a copy number (CN) of 3 at all target regions only indicates the presence of a *CYP2D6* duplication, but not if there is a CYP2D6*2x2 or *4x2 duplication. In this specific situation, knowing which allele is duplicated is necessary for therapeutic decision-making. Per the current ClinPGx/CPIC translation tables (https://www.clinpgx.org/page/cyp2d6RefMaterials; last accessed 29 September 2025), CYP2D6*2x2/*4 is classified as a normal metabolizer (Activity Score (AS) = 2.0) while a *2/*4x2 is an intermediate metabolizer (AS = 1.0). Therefore, there is a need for straightforward and reliable methods for determining which *CYP2D6* star alleles are duplicated, especially if the star alleles in the diplotypes confer differential activity. Thus, the primary goal of our study was to develop and validate a novel method, StarTRAC-*CYP2D6* (**T**argeted **R**eporting of **A**llele-specific **C**NV for *CYP2D6*) to interrogate CN status of commonly observed duplications in an allele-specific manner using the Absolute Q™ dPCR platform.

Our secondary goals were to explore (1) how the quantity of DNA input may affect the interpretation of copy number by digital PCR and (2) to what extent non-targeted variants interfere with assay performance. In previous work, 10 ng of genomic DNA was used to attain a target copy concentration between 100–500 copies/µL to conservatively maintain linearity within the Poisson distribution (Wang et al., 2024). In this study, we aimed to explore the upper limits of the distribution to allow for more flexible DNA input amounts. To our knowledge, there has been no published study examining the effect of DNA input on copy number integrity (CNI), which may be important for accommodating and optimizing CNV testing workflows. Here, we define CNI as the agreement of the calculated CN to the expected CN. To accomplish this, we tested how various levels of DNA input affected the calculated CN. CNI was preserved if this value falls within the ±0.25 confidence threshold of the expected CN value. We also provide two cases exemplifying the range of effects that non-targeted variants may have in disrupting assay design regions, i.e., interference with primer/probe binding. While there have been previous reports of variant interference for gene region CNV determination (Turner et al., 2021; Sicko et al., 2022; Davids et al., 2025), these examples demonstrate how variant interference can manifest when interpreting StarTRAC-*CYP2D6*.

## Materials and methods

### Nomenclature

*CYP2D6* star alleles are as defined by PharmVar, where core variants describe individual haplotypes. Other variants (non-coding and synonymous) then define the more granular definitions of suballeles. Variants are described using the genomic NG_008376.4 reference sequence (RefSeq), where ATG is +1, and NP_000097.3 was used to describe amino acid changes.

### Samples

A total of 17 DNA samples from the Coriell Institute for Medical Research (Camden, NJ) were used for this study. These samples were previously characterized by the Genetic Testing Reference Material Coordination Program (GeT-RM) for *CYP2D6* (Gaedigk et al., 2019) and have also been further studied by others (Choudhury et al., 2020; Wang et al., 2022; 2024; Twesigomwe et al., 2023); Coriell samples not characterized by the GeT-RM were subjected to similarly rigorous gene characterization as described in those publications. Briefly, the methods used include single-tube and OpenArray™ panel genotyping by TaqMan™, Sanger sequencing of XL-PCR amplicons, CNV as determined by Bio-Rad ddPCR and/or Absolute Q™ dPCR platforms.

### StarTRAC-*CYP2D6* assays

TaqMan™ genotyping (GT) and CN assays (Thermo Fisher Scientific, Waltham, MA) were used to determine the number of gene copies present and which star allele(s) are duplicated or have multiple copies. Commercially available TaqMan™ GT assays capture both the reference and variant alleles using FAM and VIC fluorescent dyes. An additional assay capturing the variant “C” of g.4181G>C (p.S486T; rs1135840) was custom-labeled with JUN. This single nucleotide variant (SNV) was chosen because it is part of many core star allele definitions and can serve as an additional informative CN result. RNaseP or *TERT* served as 2-copy (CN neutral) reference gene assays and were customized with an ABY dye label. All individual assays were provided by Thermo Fisher Scientific (Waltham, MA) and are described in Table 1 with their respective target(s), assay IDs, and dyes.

**TABLE 1:** Individual TaqMan^™^ GT and CN assays used in this study. GT assays target both the reference and variant allele which correspond to either FAM or VIC dyes, as displayed. Individual assay information is available on the Thermo Fisher Scientific website.

Target	rs#	Assay ID	Fluorescent dye labels^a^
g.2851C>T	rs16947	C__27102425_50	FAM > VIC
g.1847G>A	rs3892097	C__27102431_D0	VIC > FAM
g.100C>T	rs1065852	C__11484460_40	FAM > VIC
g.1022C>T	rs28371706	C__2222771_A0	VIC > FAM
g.3184G>A	rs59421388	C__34816113_20	VIC > FAM
g.2989G>A	rs28371725	C__34816116_20	VIC > FAM
g.4181C^b^	rs1135840	C__27102414_10	JUN
*TERT* ^b^	*-*	Cat # 4403316	ABY
RNaseP (*RPPH1*)^b^	*-*	Cat # 4403328	ABY

^a^ Fluorescent dyes are not consistently labeled with either the reference or variant. For example, VIC, is the variant for the g.2851C>T assay (C__27102425_50) but the reference for the g.1847G>A assay (C__27102431_D0). Information regarding dye label specifics can be found on the Thermo Fisher Scientific TaqMan™ catalogue website.

^b^ Assay with custom-ordered fluorescent dye.

**FIGURE 1:**
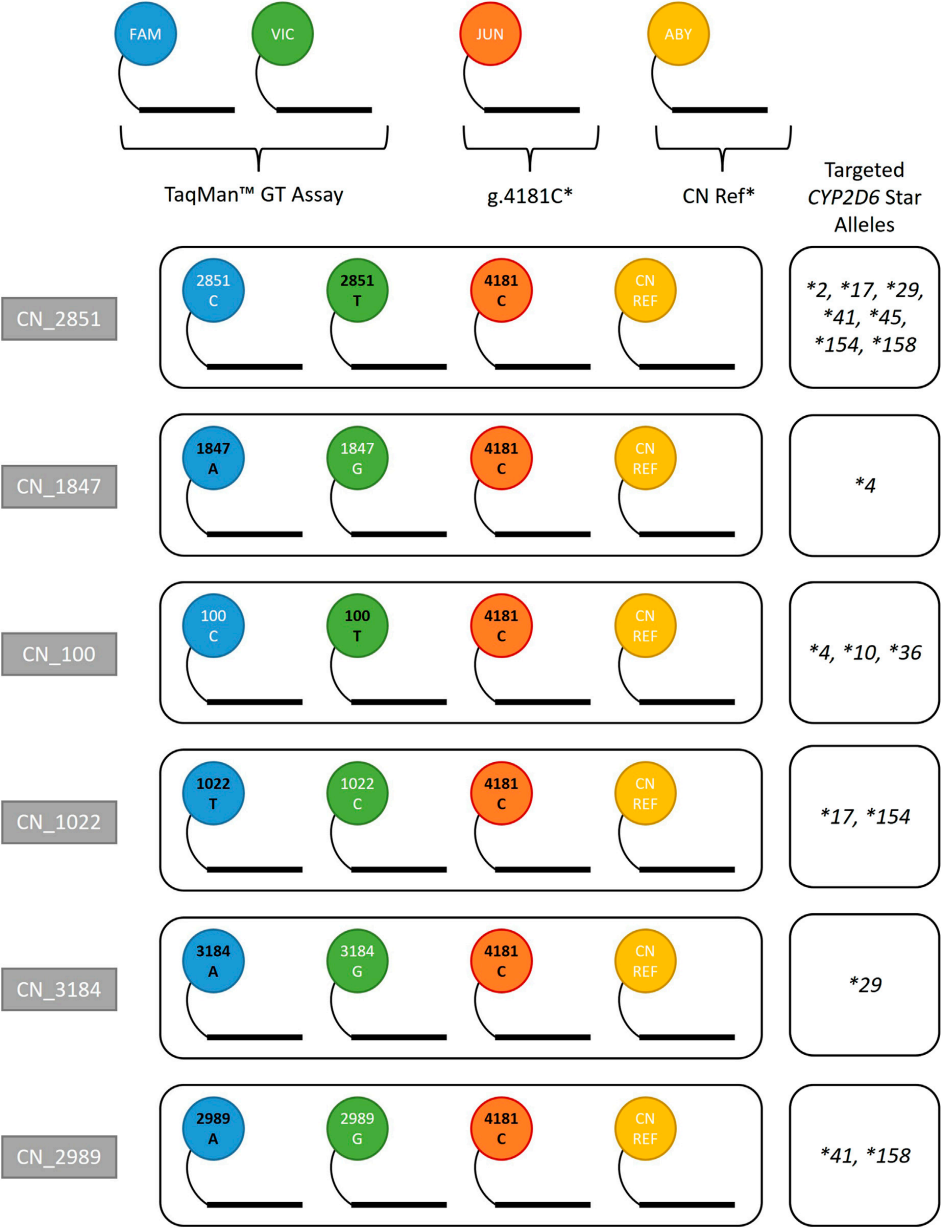
StarTRAC-*CYP2D6* multiplex assays and assay components. TaqMan™ GT assays contribute FAM and VIC probes, which were swapped in each assay combination depending on desired targeted allele(s). A custom GT assay targeting g.4181C with a JUN dye was used to capture additional information, since g.4181G>C is a core variant of many star alleles. The variant alleles in each assay are identified by black text, including g.4181C. g.4181C* and CN Ref* denote custom-ordered assays. The “CN Ref” assays used were either *TERT* or RNaseP. The star alleles targeted in this study are displayed for each assay combination for reference; however, these assays may also be able to discriminate other alleles.

Figure 1 summarizes the 4-plex assay combinations designed for this study. Each combination targets core SNVs found in commonly duplicated alleles. The format for each assay combination contains a TaqMan™ GT assay (FAM/VIC), g.4181C (JUN), and reference gene assay (RNaseP or *TERT* labeled with ABY). RNaseP was used as the default reference gene assay while *TERT* was validated as an alternative reference for confirmatory purposes (data not shown). Assay combinations are referred to as CN_2851, CN_1847, CN_100, CN_1022, CN_3184, and CN_2989 (the number represents the position of the interrogated SNV on the genomic RefSeq). Each StarTRAC-*CYP2D6* 4-plex assay is differentiated by the TaqMan™ GT assay used to target a specific core variant. The CN status of this core variant is then used to interpret the CN status of associated star allele(s). For example, CN_2851 contains the GT assay targeting g.2851C>T, which is part of the CYP2D6*2, *17, *29, and many other star alleles.

### StarTRAC-*CYP2D6*

Allele-specific copy number determination was performed on the Applied Biosystems™ QuantStudio™ Absolute Q™ dPCR System. The single-step, one-pot restriction enzyme digestion workflow was used with Anza™ 69 *Bgl*I (Invitrogen, Waltham, MA) as previously described (Wang et al., 2024). In short, a 10 µL reaction mix was combined using nuclease free water, 10 ng genomic DNA, 2.5 U/µL of *Bgl*I, and the following reagents with their final concentrations: 0.25x Anza Clear Buffer, 1x TaqMan™ GT Assay, 1x g.4181C custom TaqMan™ assay, 1x gene reference assay, and 1x Absolute Q DNA dPCR Mix. From this reaction mix, 9 µL were transferred into a well of the MAP16 plate and 15 µL of Isolation Buffer were layered on top. After a 30-min benchtop incubation (for genomic DNA digestion), the MAP16 plate was loaded on the Absolute Q™ for cycling and signal detection. The cycling protocol is as follows: 1 cycle of 96 °C for 10 min, 40 cycles of 96 °C for 5 s and 60 °C for 15 s.

### DNA input and CN integrity (CNI) study

To examine the effect of DNA input on CNI (*e.g.*, if calculated CN is consistent with expected CN), Coriell samples were tested as previously described (Wang et al., 2024). Collectively, four Coriell samples represent CN = 1, 2, 3, 4, and 6; one of the samples has complex structural variation and represents two copy number states (CN = 3 and CN = 6). Each sample was subjected to the *CYP2D6* CN triplex assay (5′UTR, intron 6, and exon 9 gene region targets) using *TERT* as the 2-copy reference gene assay. Coriell IDs and expected CN status at each target region are provided in Supplementary Table S1. DNA input was determined by making seven serial dilutions from the Coriell DNA stock. Two µL of each serial dilution (1:2, 1:4, 1:8, 1:16, … , etc.) and the stock were individually combined with the reaction mixture and run on the Absolute Q™. Total DNA input was estimated by taking a spectrophotometers measurement (NanoDrop One; Thermo Fisher Scientific, Waltham, MA) of each dilution.

### Data analysis

Run data were analyzed using the Applied Biosystems™ QuantStudio™ Absolute Q™ Digital PCR Software (version 6.2.1 and 6.3.0). Each sample was manually inspected for proper cluster separation in the four fluorescent channels using both 1D and 2D views. Examples are provided in the Supplementary Figure S1. For both the DNA input study and the StarTRAC-*CYP2D6* validation, calculated CN was considered valid if it was within ±0.25 of the expected integer value. For example, a CN call of 2 was determined if the calculated CN was between 1.75 and 2.25.

## Results

**FIGURE 2:**
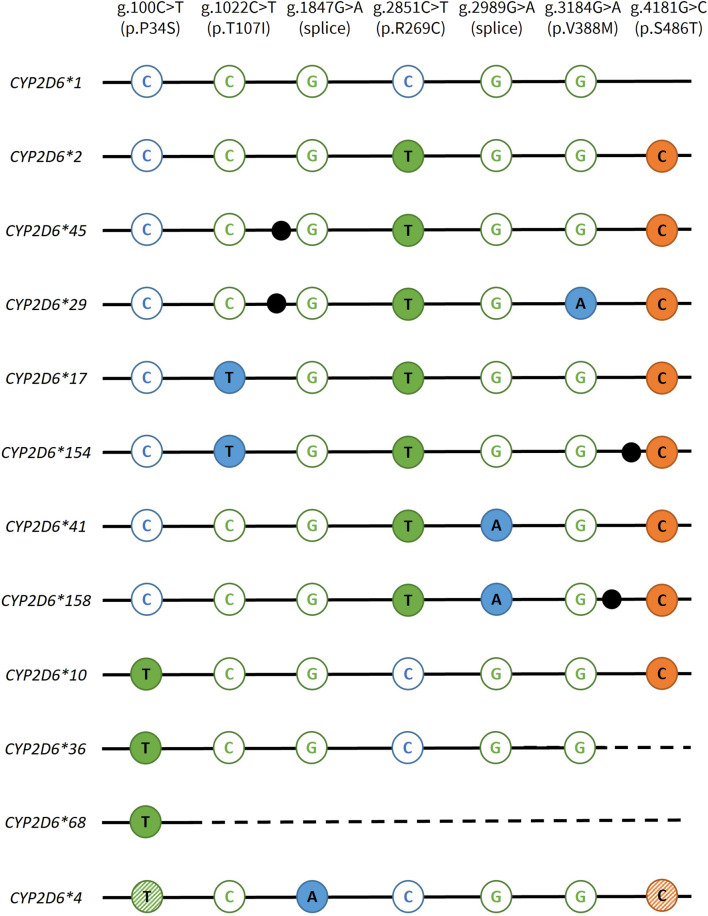
Overview of the genotyping targets used to discriminate *CYP2D6* star alleles. *CYP2D6* genotyping variants are specified in the top row. FAM targets are displayed in blue, VIC in green, and JUN in orange. Open and solid circles represent assays detecting the reference and variant alleles, respectively. For CYP2D6*4, the hatched circles indicates that these variants are present on most but not all suballeles. Depending on which suballele is present, these variants may or may not be informative. Dotted lines indicate gene conversions to *CYP2D7*, which are not amplified by the *CYP2D6-*specific assays. Black dots indicate the presence of an additional core SNV that are not targeted in this study: CYP2D6*45, g.1717G>A, p.E155K; CYP2D6*29, g.1660G>A+g.1662G>A, p.V136I; CYP2D6*154, g.4046G>A, p.R411H; CYP2D6*158, g.3187A>C, p.I339L.

For our primary objective, a total of 17 Coriell samples with well-characterized genotypes and CNV status were tested to validate the StarTRAC-*CYP2D6* multiplex method. All allele-specific StarTRAC-*CYP2D6* CN calls were consistent with their previously established diplotypes for copy neutral (CN = 2) as well as for duplication/multiplication, and hybrid containing samples. These results demonstrated that the StarTRAC-*CYP2D6* method is a robust means of determining star allele duplications. Figure 2 visualizes the *CYP2D6* GT targets for all star alleles tested in this study. For the secondary objectives, CNI was maintained for CN status up to 6 copies when total DNA input was below 200 ng. Interfering variants were observed in one case where a non-targeted SNV did not affect CN determination and one case where a non-targeted SNV caused a drop-out in signal, *i.e.*, the calculated CN signal of the targeted SNV fell below the valid threshold.

### Validation of genotyping targets

Five copy-neutral DNA samples were tested to validate the allele-specificity of each StarTRAC-*CYP2D6* multiplex. All allele-specific CN determinations were in concordance with their consensus diplotypes as summarized in Table 2. NA17111 (CYP2D6*1/*1) was used as a homozygous reference control for all StarTRAC-*CYP2D6* multiplexes and results were consistent with 2 copies for all reference targets and 0 copies for all variant targets. Heterozygous control samples were also tested by relevant StarTRAC-*CYP2D6* assays to confirm simultaneous detection of both the reference and variant SNV targets. Each sample produced 1 copy of the reference and 1 copy of the variant. For example, NA18966 (CYP2D6*1/*2) assayed with CN_2851 yielded 1 copy of g.2851C (reference) and 1 copy of g.2851T (variant).

**TABLE 2:** Summary of starTRAC-*CYP2D6* CN calls obtained for copy neutral, CN = 2, Coriell DNA samples.

		NA17111 (*1/*1)		NA18966 (*1/*2)		NA06991 (*1/*4)		HG03313 (*29/*154)		NA19316 (*2/*158)	
4-plex	Target	REF	VAR	REF	VAR	REF	VAR	REF	VAR	REF	VAR
CN_2851	g.2851C>T	2	0	1	1	-	-	-	-	-	-
CN_1847	g.1847G>A	2	0	-	-	1	1	-	-	-	-
CN_100	g.100C>T	2	0	-	-	1	1	-	-	-	-
CN_1022	g.1022C>T	2	0	-	-	-	-	1	1	-	-
CN_3184	g.3184G>A	2	0	-	-	-	-	1	1	-	-
CN_2989	g.2989G>A	2	0	-	-	-	-	-	-	1	1
All	g.4181C	-	0	-	1	-	1	-	2	-	2

REF denotes CN of the reference allele and VAR denotes CN of the variant allele. g.4181C was tested as part of each 4-plex and resulting CN calls are shown as the consensus of the assays. ‘-’ indicates that sample was not tested for the specified assay.

### Duplications/multiplications

**FIGURE 3:**
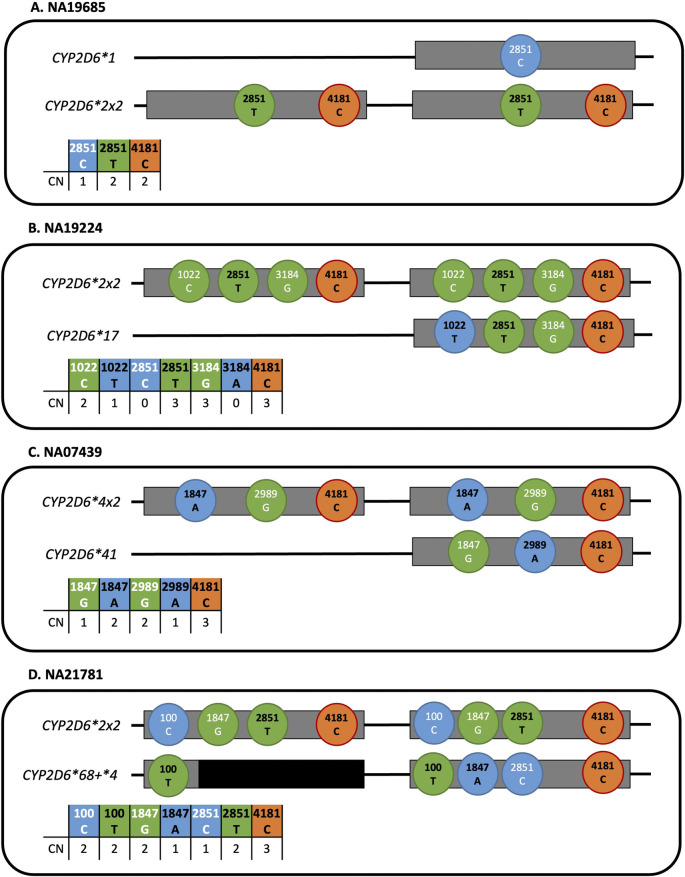
Visualization of the targets for the StarTRAC-*CYP2D6* multiplex reactions as illustrated on representative samples. Grey bars represent *CYP2D6* gene copies. Colored circles represent the assay’s associated dye channel: blue (FAM), green (VIC), and orange (JUN). Black and white text in circles indicate whether the variant or reference allele is targeted, respectively. CN results for each individual sample and target are concurrently displayed. **(A)** NA19685 (CYP2D6*1/*2x2) was tested with CN_2851 and showed two copies of the *2 core variant indicating a *2x2 duplication. **(B)** NA19224 (CYP2D6*2x2/*17) shows an example where only one assay, CN_1022, is informative for determining allele-specific CN. Detecting two copies of g.1022C and one copy of g.1022T, indicates presence of a *2x2 duplication. **(C)** In contrast, NA07439 (CYP2D6*4x2/*41) represents an example where two CN multiplex reactions, either CN_1847 or CN_2989, were informative for determining *4x2 duplication status. **(D)** NA21781 (CYP2D6*2x2/*68+*4) represents a sample with a hybrid gene; *CYP2D7-*derived regions are displayed in black. Assays targeting g.1847G>A, g.2851C>T, and g.4181C do not produce signal from the *CYP2D7*-derived portions of the CYP2D6*68 gene copy due to *CYP2D6* sequence specificity.

The efficiency of the restriction enzyme digest and ability of the 4-plex StarTRAC-*CYP2D6* multiplex to capture duplications was confirmed by testing NA19685 with CN_2851 (Table 3; Figure 3A). Based on the consensus diplotype, CYP2D6*1/*2x2, the *1 contributed 1 copy of g.2851C (reference allele) and *2x2 contributed 2 copies of g.2851T (variant allele) and g.4181C (variant allele). The CYP2D6*2 is interpreted as being the duplicated allele because there were 2 copies of the two core variants defining the *2 allele.

**TABLE 3:** Summary of starTRAC-*CYP2D6* CN calls for coriell samples known to have a structural variation (duplication, multiplication, and/or hybrid).

		CN_2851		CN_1847		CN_100		CN_1022		CN_3184		CN_2989		CN_4181
Sample	Consensus diplotype	C	T	G	A	C	T	C	T	G	A	G	A	C
NA19685	*1/*2x2	1	2	-	-	-	-	-	-	-	-	-	-	2
NA19920	*1/*4x2	3	0	1	2	1	2	-	-	-	-	-	-	2
NA24217	*2/*41x3	0	4	-	-	-	-	-	-	-	-	1	3	4
NA19224	*2x2/*17	0	3	-	-	-	-	2	1	3	0	-	-	3
NA19109	*2x2/*29	-	-	-	-	-	-	-	-	2	1	-	-	3
NA17113	*17x2/*45	-	-	-	-	-	-	1	2	-	-	-	-	3
NA07439	*4x2/*41	-	-	1	2	-	-	-	-	-	-	2	1	3
NA23297	*10x2/*17	-	-	-	-	1	2	2	1	-	-	-	-	3
NA21781	*2x2/*68+*4	1	2	2	1	2	2	-	-	-	-	-	-	3
NA23246	*10x2/*36+*10	-	-	-	-	0	4	-	-	-	-	-	-	3

The variant alleles are underlined for each targeted assay. g.4181C (variant) is part of each 4-plex reaction and CN calls represent the consensus of the assays. ‘-’ indicates that sample was not tested for the specified assay.

Seven other samples with duplications or multiplications were subsequently chosen to validate StarTRAC-*CYP2D6*. As summarized in Table 3, the assays captured allele-specific CNs between 0–4 which aligned with each sample’s consensus diplotype. These samples were tested with multiple StarTRAC-*CYP2D6* multiplexes to demonstrate the importance of choosing an informative GT target. For example, NA19224 (CYP2D6*2x2/*17) was run with CN_2851, CN_1022, and CN_3184; however, only CN_1022 was informative because g.1022C>T was the only variant that differentiated between *2 and *17. As visualized in Figure 3B, the CYP2D6*2x2 duplication contributed 2 copies of g.1022C and the *17 allele contributed 1 copy of g.1022T. In contrast, CN_2851 and CN_3184 were not informative because their respective GT targets were either all present or absent in the gene copies: CN_2851 yielded 0 copies of g.2851C and 3 copies of g.2851T while CN_3184 yielded 3 copies of g.3184G and 0 copies of g.3184A.

Other samples demonstrated situations where more than one StarTRAC-*CYP2D6* multiplex may be informative for interpreting which star allele is duplicated. Figure 3C shows NA07439 (CYP2D6*4x2/*41) which was tested with CN_1847 and CN_2989 targeting g.1847G>A and g.2989G>A, respectively. Both GT targets could be used to differentiate between the CYP2D6*4 and *41 alleles and thus determine which one is duplicated. CN_1847 resulted in 1 copy of g.1847G from the CYP2D6*41 allele and 2 copies of g.1847A from the *4 allele indicating that the latter is duplicated. For CN_2989, the CYP2D6*4 duplication yielded 2 copies for g.2989G, and the single *41 allele yielded 1 copy for g.2989A.

Gene duplications greater than 2 copies, could also be detected in an allele-specific manner. NA24217 (CYP2D6*2/*41x3) was tested with CN_2989, producing 1 copy for g.2989G and 3 copies of g.2989A which is consistent with a CYP2D6*41x3 multiplication.

### Hybrids

Two additional Coriell samples were tested with StarTRAC*-CYP2D6* multiplexes to evaluate performance of complex structures containing hybrid genes. NA21781 (CYP2D6*2x2/*68+*4) has an identical duplication on one allele and a nonidentical gene duplication on the other, of which the *68 gene copy is a *CYP2D6::CYP2D7* hybrid. The calls produced by the CN_2851, CN_1847, and CN_100 assays were each 3 copy and concordant with the diplotype, as no CN signals were produced from the *CYP2D7-*derived portion of the CYP2D6*68 hybrid (Table 3; Figure 2). Figure 3D illustrates this result being consistent with CYP2D6*68 converting from *CYP2D6* to *CYP2D7* after intron 1. *CYP2D6*-specific signal generation is demonstrated by testing with CN_100, which produced a total of 4 copies, *i.e.*, 2 copies of g.100C and 2 copies of g.100T. Accurate detection of *CYP2D6*-specific regions within a gene copy were also demonstrated on NA23246 (CYP2D6*10x2/*36+10) with the CN_100 assay. Four gene copies were detected for g.100T, but only 3 copies for g.4181C. Here, the *CYP2D7*-dervived exon 9 region within the CYP2D6*36 hybrid caused a dropout of the g.4181C assay signal.

### Interpretation of g.4181C assay results

In the examples described above and illustrated in Figures 3B–D, the g.4181C assay alone cannot be used to interpret the duplicated star allele because all gene copies tested contain g.4181C. In these instances, the g.4181C assay can serve as an internal *CYP2D6* copy-control to capture the total number of gene copies with g.4181C. In certain situations, however, this target may provide additional or confirmatory information for samples that are heterozygous for this variant, as visualized in Figure 3A. Another example is NA19920 (CYP2D6*1/*4x2) (Table 3). Here, 2 copies of g.4181C was interpreted as the non-CYP2D6*1 gene copy being duplicated. Of the targetable variants present, g.4181C can also be used as another differential variant amongst the three gene copies.

### DNA input and copy number integrity (CNI)

**FIGURE 4:**
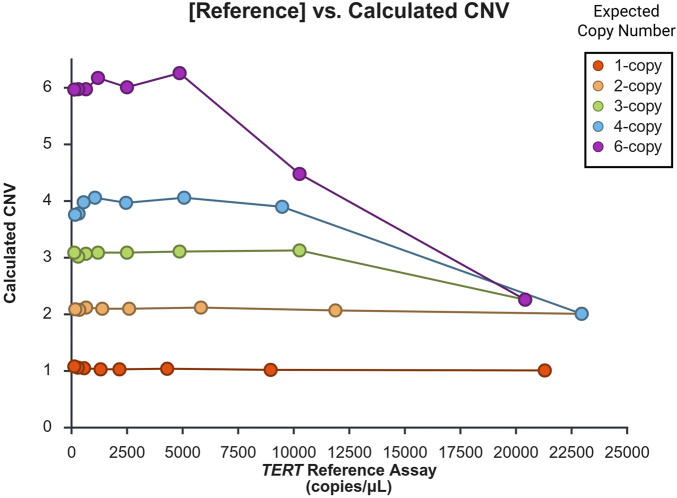
The impact of DNA input on assay performance. This graph displays the relationship between the amount of input DNA used in each reaction and the calculated CN state measured for different *CYP2D6* gene regions (5′UTR, intron 6, or exon 9). *TERT* was used as the 2-copy reference gene assay. Coriell sample IDs used are provided in Supplementary Table S1, and Supplementary Table S2 provides targets, calculated CN, and copies/µL. DNA input of 200 ng was optimal across the represented CN status, as higher input may affect CN call integrity.

Four Coriell samples representing CN = 1, 2, 3, 4, and 6 were tested using serial dilutions of the DNA stocks. Figure 4 graphically displays the amount of input DNA (ng) and resulting calculated CN values for each expected CN. For 1-copy and 2-copy samples, CNI was maintained throughout the dilution series up to the maximum amount of about 1 µg DNA. For the 3-copy and 4-copy samples, CNI begins to decay between 350 and 450 ng and for the 6-copy sample, the CNI begins to decay around 200 ng. For the higher amounts of DNA input, the CN trends towards 2 copy calls. Because the calculated CN is also determined by the concentration ratio of [target] to [reference], we examined these individual metrics in relation to CNI as well (Supplementary Figure S2; Supplementary Table S2). When concentrations (copies/µL) were plotted against calculated CN, trends similar to those found in the DNA input experiment were observed, *i.e.*, higher CN values were affected by CN decay at lower concentrations than lower CN values; the decay also trended towards an erroneous 2-copy interpretation. Conservatively, CNI was maintained for 6 copies when the [target] was below 17,500 copies/µL and 5,000 copies/µL for the [reference]. For 4 copies and below, CNI was consistently preserved up to 20,000 copies/µL of [target] and 10,000 copies/µL of [reference].

### SNV assay interference

#### Reference gene assay

**FIGURE 5:**
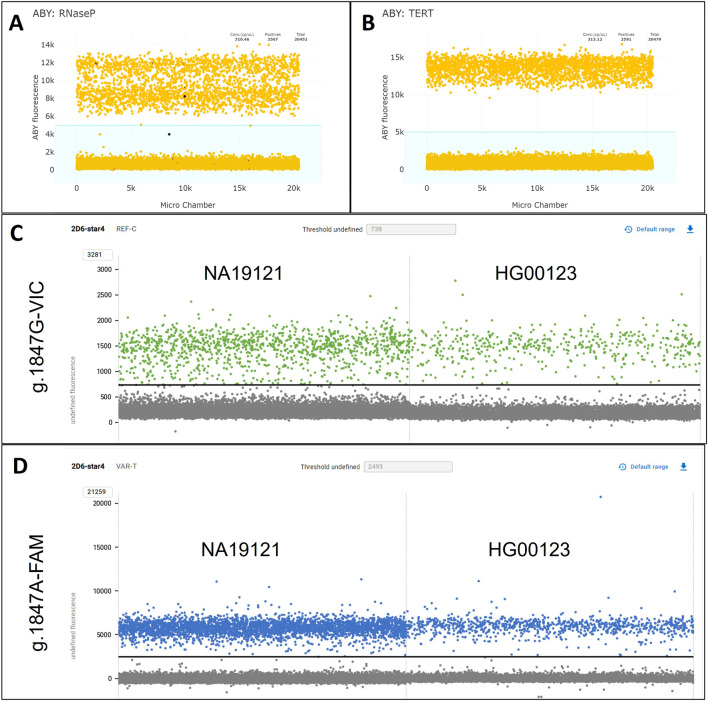
1D-plots of samples with assay-interfering variants. **(A,B)** Displays the ABY-reference gene channel for HG03313 (CYP2D6*29/*154) tested with CN_1022 with RNaseP **(A)** and *TERT* **(B)** as reference genes. The light blue shaded areas visualize the manual gating used for assay interpretation. **(C)** Shows the VIC dye channel targeting g.1847G of NA19121 (CYP2D6*1.068/*4x2) and HG00123 (*1.005/*4.004) tested with CN_1847, where the amplitude was lower than expected and more scattered due to g.1870T>C interfering with the assay. **(D)** Shows the same samples as **(C)** but in the FAM channel targeting g.1847A, where the amplitude and clustering were unaffected by g.1870T>C.

Testing HG03313 (CYP2D6*29/*154) with CN_1022 and RNaseP as the gene reference assay produced an unexpected double-positive cluster for the ABY fluorescent channel (Figure 5A). While this anomaly was visually distinctive, it did not push the calculated CN results outside of the valid threshold with proper gating of negative and positive reactions (Supplementary Table S3). When using *TERT* as the reference gene assay (Figure 5B), the ABY channel produced a single, cohesive positive cluster and CN results were consistent with those obtained with RNaseP. To explore possible causes of the double-positive ABY cluster in HG03313, high quality whole genome sequencing data available from the 1000 Genomes Project were searched for potential variants within the targeted RNaseP (*RPPH1)* gene region. Variant rs3093876 was identified and confirmed to interfere with the RNaseP assay (correspondence with Thermo Fisher Scientific).

#### Genotyping (GT) assay

Two Coriell samples, HG00123 (CYP2D6*1.004/*4.004) and NA19121 (CYP2D6*1.068/*4x2), were confirmed by Sanger sequencing to harbor a synonymous variant, g.1870T>C (rs111606937) on the *1 allele. This SNV interfered with the TaqMan™ GT assay targeting the CYP2D6*4 core variant, g.1847G>A (rs3892097); Supplementary Figure S3 displays how the allelic discrimination plot from a traditional single-tube TaqMan™ GT assay (C__27102431_D0) is affected by this interference, which resulted in a false g.1847A/A interpretation, due to the variant disrupting g.1847G (reference) primer/probe binding (correspondence with Thermo Fisher Scientific). To investigate how g.1847G>A may affect StarTRAC-*CYP2D6*, both samples were run with CN_1847 and CN_100 (Table 4). The calculated CN for g.1847G target (reference allele) was below the valid threshold for making a valid CN call for both samples, 0.48 for HG00123 and 0.62 for NA19121. The visualization in the dye channels (Figures 5C,D) also reflected this interference, where the positive clusters in g.1847G (reference) VIC channel, reached an amplitude of approximately 1,500 RFUs, which was considerably lower than the positive clusters in the g.1847A (variant) FAM channel which approximated 6,000 RFUs. Notably, the clustering for the positive reactions in the VIC channel appeared more disperse, which further supports a disruption in assay binding efficiency.

**TABLE 4:** Calculated CN results for HG00123 and NA19121 tested with StarTRAC-*CYP2D6*.

Sample	Consensus Diplotype	CN_1847			CN_100		
		G	A	g.4181C	C	T	g.4181C
HG00123	(*1.005/*4)	0.48	1.00	0.93	0.98	1.00	0.98
NA19121	(*1.068/*4x2)	0.62	2.05	1.91	1.00	1.95	1.97

Both samples have g.1870T>C (rs111606937) that interferes with the assay measuring g.1847G. Variant alleles are underlined. Here, the calculated CN is displayed instead of the CN call to show that the results for g.1847G are outside the range for valid CN determination. For both samples, calculated CN for g.1847G is expected to fall between 1.25–0.75 as there is one copy of g.1847G.

## Discussion

In this study, we developed a novel method for allele-specific copy number determination, StarTRAC-*CYP2D6*. Building on a previous strategy of optically multiplexing three *CYP2D6* target regions for dPCR (Wang et al., 2024), we accomplished our primary goal of reliably determining the CN status of *CYP2D6* core variants to interpret star allele duplications/multiplications. Experiments with copy neutral (CN = 2) Coriell DNA samples demonstrated allele-specificity of assays interrogating seven *CYP2D6* core variants that are commonly found in SVs: g.2851C>T, g.1847G>A, g.100C>T, g.1022C>T, g.3184G>A, g.2989G>A, and g.4181C. Additionally, we demonstrated that CN integrity is preserved for CN states up to 6 copies when DNA input is limited to 200 ng per reaction and the reference assay concentration is under 5,000 copies/µL. Furthermore, we present cases that are helpful for trouble-shooting unexpected findings caused by the presence of uncommon interfering SNVs. A variant in *RPPH1* (rs3093876) caused a double positive cluster to appear in the 1D view of the ABY dye channel. Although this presentation did not affect CN determination with proper gating, substituting the reference assay with *TERT* resolved the artifact. In another case, a *CYP2D6* variant, g.1870T>C (rs111606937), compromised the calculated CN for the g.1847G target of CN_1847, resulting in a value outside the valid range for call determination.

### StarTRAC-*CYP2D6*

The application of StarTRAC-*CYP2D6* is most valuable for cases when phenotype prediction is dependent on which allele exhibits an SV. For example, StarTRAC-*CYP2D6* can easily discriminate whether an individual is a CYP2D6*2x2/*4 (normal metabolizer) or *2/*4x2 (intermediate metabolizer) or CYP2D6*1/*41x3 (normal metabolizer) or *1x3/*41 (ultrarapid metabolizer). With the set of TaqMan™ GT assays used in this study, commonly observed duplications such as CYP2D6*1xN, *2xN, *4xN, *10xN, *17xN, *29xN and *41xN can be discriminated. Additional duplications (*e.g.*, CYP2D6*3xN, *9xN, *etc*.) can be readily detected by applying the StarTRAC-*CYP2D6* method with other genotyping assays, as long as other method requirements are met–such as ensuring the restriction enzyme does not cut within assay amplicons. Although commercially available TaqMan™ GT assays that detect both reference and variant alleles were primarily used, custom combinations targeting only the variant alleles offer an alternative approach to maximize detection across three distinct SNV targets and a single reference gene – one for each dye channel. For example, multiplexing g.2851T-FAM, g.100T-VIC, g.2989A-JUN, and reference gene-ABY covers three SNVs present in several *CYP2D6* decreased and no function alleles. However, an important caveat with only testing the variant alleles is being able to discern if a lack of signal is due to the absence of the variant allele or the presence of *CYP2D7-*derived sequences as demonstrated with the g.4181C assay on samples with a *CYP2D6::CYP2D7* hybrid gene (Table 3; Figure 3D). Another potential reason for an unexpected loss of signal is if a targeted variant is triallelic; an example of this scenario is CYP2D6*8 which has g.6778G>T and *14 which has g.6778G>A. Additionally, lack of signal could also be due to the interrogated *CYP2D6* sequence harboring other rare and/or unknown SNVs that prevent amplification or interfere with assay probe binding, such as the interferences observed in this study (Table 4; Figure 5).

### StarTRAC-*CYP2D6* assay limitations

Beyond other considerations for assay design (Whale et al., 2016), limitations include the necessity for custom dye labels for multiplexing, which may incur additional costs, and SNVs of interest may not have commercial TaqMan™ assays available. Additionally, access to a dPCR instrument with fluorescent channel multiplexing capabilities is also required. As with any PGx genotyping platform, success can also be limited by user expertise and knowledge of pharmacogene allele complexity, especially *CYP2D6.* As with all genotyping assays, the presence of rare or novel SNVs may also interfere with results and data interpretation as further discussed below. Finally, StarTRAC-*CYP2D6* does not provide information on the phase of gene copies, for example, does not determine whether the sample is a CYP2D6*10x2/*36+*10 or *10x3/*36. However, this limitation does not affect the overall phenotype prediction.

### DNA input and copy number integrity (CNI)

Based on our findings the amount of DNA should not exceed 200 ng per reaction or greater than 5,000 copies/µL of the [reference] to maintain CNI up to 6 copies. However, because 6 copies of *CYP2D6* is a rare occurrence, up to 450 ng of DNA may be applied to capture samples up to 4 copies. This may be advantageous for optimizing laboratory workflows, as an additional dilution step may be omitted after DNA extraction and before dPCR set-ups. Furthermore, we recommend keeping [reference] under 10,000 copies/µL to ensure CNI for most samples. When the [reference] is over 10,000 copies/µL, users should be discerning for high CN values that fall outside the linearity of the Poisson distribution. This may be attributed to the oversaturation of the microchambers with positive reactions and explain why the higher CN controls (CN = 4 and 6) trend towards 2 copies as the amount of DNA input increases; the gene reference is 2 copy. In these situations, a valid CN calculation may be achieved by repeating the assay with diluted DNA and/or using a CN reference assay greater than 2 copy. Although our experiments were limited in repetitions and the range of DNA input examined, our findings can inform other laboratories to optimize their workflow for their own testing.

### Interfering SNVs

The cases described in this report are consistent with previous observations of variants interfering with CN determination using a digital PCR platform. Effects can range from benign artifacts to disrupting CN calls or genotyping interpretations.

We have previously described a CNV in a reference gene, i.e., a *TERT* gene duplication, which resulted in proportionally skewing the calculated CN result. This was resolved by repeating the sample with a different reference gene assay, RNaseP (Wang et al., 2024). We investigated rs3093876 in the RNaseP reference gene (*RPPH1)*, which was previously reported to interfere with CN calling using qPCR (Sicko et al., 2022). Here, dPCR-based CN calls were unaffected, but the aberrant double clustering could potentially be gated incorrectly and lead to inaccurate CNV determination or indeterminate calls. Because RNaseP is commonly used as a reference gene for CNV testing, awareness of this phenomenon is necessary for users to accurately analyze the cluster plots; rs3093876 is most often observed in individuals of African ancestry (1.4%) while the total SNV frequency is 0.15% (https://gnomad.broadinstitute.org/variant/14-20343365-G-A?dataset=gnomad_r4; accessed 17 October 2025).

More impactful are the cases where calculated CN falls outside the range to make valid CN calls. For HG00123 and NA19121, we observed a dropout in the signal for the reference allele, g.1847G. The calculated CN was 0.48 and 0.62, respectively, which is below the 1.25–0.75 valid threshold for a 1-copy sample. This drop in signal was due to the presence of a synonymous variant, g.1870T>C (rs111606937) that interfered with primer/probe binding. This variant is present in two CYP2D6*1 suballeles, CYP2D6*1.005 and *1.068, the latter being discovered in this study. The novel CYP2D6*1.068 suballele was fully characterized and submitted to PharmVar for allele designation. According to gnomAD (v.4.1.0), g.1870T>C occurs at a global allele frequency of 0.23% and is most commonly found in non-Finnish Europeans (0.25%) and people of African ancestry (0.36%) (https://gnomad.broadinstitute.org/variant/22-42128922-A-G; accessed 17 October 2025).

These examples highlight the presence of a non-targeted SNV that can result in a CN falling outside of the expected range. Other reasons include poor DNA quality, inappropriate restriction enzyme selection, insufficient restriction enzyme digestions, *etc*. While this is the first example of variant interference for allele-specific CN determination, other *CYP2D6* variants/haplotypes have been reported to interfere with CN determination (Turner et al., 2021). Although it is impossible to account for all variants in the highly polymorphic *CYP2D6* gene to avoid interferences when designing assays, it is still valuable to characterize underlying culprits of unexpected results, as this may facilitate troubleshooting. Based on our experience, we emphasize the importance of manually examining data for proper cluster separation and considering the use of an alternative reference gene assay for follow-up testing. For StarTRAC-*CYP2D6*, solutions may include re-designing genotyping assays around known interfering variants or using another differential target variant if possible. Redesigns from Thermo Fisher Scientific are currently underway for the RNaseP reference gene assay and the TaqMan GT assay targeting g.1847G>A to avoid the interference of the SNVs observed in this study.

For future method development, protocols may integrate *CYP2D6* CN and allele-specific CN determination as they can both be carried out on dPCR platforms with higher-level multiplexing capabilities. The simultaneous detection of gene region CN and allele-specific CN would further streamline pharmacogenetic testing reducing the need for additional or reflex testing runs.

## Conclusion

As digital PCR is increasingly used for assessing copy number of *CYP2D6*, we developed and validated a method for resolving the allele specificity of duplications/multiplications. StarTRAC-*CYP2D6* uses a simple workflow, which can easily be expanded to include other *CYP2D6* targets or be adapted to other genes of interest. Additionally, presenting and discussing cases with unexpected results and investigations of DNA quantity impact on assay performance also provide highly valuable technical insights for dPCR users.

## Data Availability

The datasets presented in this study can be found in online repositories. The names of the repository/repositories and accession number(s) can be found below: https://www.pharmvar.org/, *CYP2D6*1.068*.
