# Supplementary material for: StarTRAC-CYP2D6: a method for CYP2D6 allele-specific copy number determination using digital PCR
Source: Front Pharmacol. 2025 Dec 2;16:1715830. doi: 10.3389/fphar.2025.1715830 (PMC12706154; doi:10.3389/fphar.2025.1715830)
Supplement: Supplementary file 1 [file DataSheet1.pdf]

Supplementary Material

1 Supplementary Figures and Tables

1.1 Supplementary Table 1

	CYP2D6 5' UTR	*CYP2D6* Intron 6	*CYP2D6* Exon 9
NA18107	6	6	3
NA24217	4	4	4
NA17111	2	2	2
NA18933	1	1	1

Expected CN for each targeted *CYP2D6* gene region assessed in the DNA input study. The three gene regions were tested simultaneously in a triplex reaction, including *TERT* as the 2-copy reference gene assay. The triplex reaction has previously been described in detail elsewhere (Wang et al., 2024).

1.2 Supplementary Table 2

			CYP2D6 5' UTR JUN		*CYP2D6* Intron 6 FAM		*CYP2D6* Exon 9 VIC		*TERT* ABY
**Sample ID**	**Dilution**	**DNA (ng)**	**Copies/µL**	**Calculate CN**	**Copies/µL**	**Calculated CN**	**Copies/µL**	**Calculated CN**	**Copies/µL**
NA18107	Stock	760.4	22978.51	2.25	22978.51	2.25	22978.51	2.25	20435.42
NA18107	1:2	362.0	22979.3	4.47	22979.3	4.47	16044.73	3.12	10283.01
NA18107	1:4	178.0	15254.08	6.25	15254.08	6.25	7570.36	3.10	4885.18
NA18107	1:8	87.4	7540.41	5.99	7564.16	6.01	3880.14	3.08	2517.09
NA18107	1:16	40.2	3756.51	6.19	3723.52	6.14	1868.69	3.08	1212.86
NA18107	1:32	24.0	1994.19	5.94	2010.31	5.99	1026.08	3.06	671.67
NA18107	1:64	12.4	957.2	5.93	968.69	6.00	485.28	3.01	322.78
NA18107	1:128	8.2	439.84	5.94	442.57	5.98	228.07	3.08	148.11
NA24217	Stock	937.8	22976.81	2.00	22976.81	2.00	22976.81	2.00	22976.81
NA24217	1:2	461.8	18830.58	3.96	18473.75	3.89	18164.65	3.82	9498.90
NA24217	1:4	223.6	10555.49	4.15	10314.91	4.05	10115.15	3.97	5090.55
NA24217	1:8	109.2	4959.36	4.01	4903.07	3.96	4885.23	3.95	2475.95
NA24217	1:16	54.8	2179.45	4.05	1997.92	3.71	2214.58	4.11	1077.14
NA24217	1:32	29.4	1126.96	3.97	1128.98	3.97	1140.8	4.01	568.44
NA24217	1:64	14.8	641.93	3.77	637.31	3.74	657.5	3.86	340.59
NA24217	1:128	6.4	338.69	3.75	339.22	3.75	348.14	3.85	180.82
NA17111	Stock	955.8	22979.07	2.00	22979.07	2.00	22979.07	2.00	22979.07
NA17111	1:2	462.0	12228.51	2.06	12015.99	2.02	12319.3	2.07	11897.26
NA17111	1:4	223.4	6145.04	2.10	6156.32	2.11	6215.22	2.13	5844.00
NA17111	1:8	108.2	2727.04	2.09	2733.66	2.09	2774.91	2.12	2615.55
NA17111	1:16	54.8	1465.22	2.09	1445.93	2.06	1478.25	2.10	1404.92
NA17111	1:32	28.2	716.98	2.11	709.13	2.08	732.92	2.15	680.27
NA17111	1:64	14.4	383.82	2.06	385.83	2.07	386.09	2.08	372.11
NA17111	1:128	6.2	200.45	2.08	194.05	2.01	205.88	2.14	192.69

Supplementary Table 2 (continued)

			CYP2D6 5' UTR JUN		*CYP2D6* Intron 6 FAM		*CYP2D6* Exon 9 VIC		*TERT* ABY
**Sample ID**	**Dilution**	**DNA (ng)**	**Copies/µL**	**Calculated CN**	**Copies/µL**	**Calculated CN**	**Copies/µL**	**Calculated CN**	**Copies/µL**
NA18933	Stock	849.2	10704.22	1.00	10284.15	0.96	10927.21	1.03	21317.57
NA18933	1:2	412.0	4539.77	1.01	4473.75	1.00	4534.13	1.01	8982.10
NA18933	1:4	205.8	2236.52	1.03	2219.63	1.03	2226.43	1.03	4326.55
NA18933	1:8	101.6	1115.82	1.02	1107.41	1.02	1131.43	1.04	2180.25
NA18933	1:16	50.0	676.62	1.02	675.87	1.02	679.81	1.02	1331.02
NA18933	1:32	25.6	304.87	1.04	311.59	1.06	304.36	1.04	587.62
NA18933	1:64	12.6	159.96	1.05	158.99	1.05	151.73	1.00	304.29
NA18933	1:128	6.6	79.35	1.07	81.7	1.10	79.24	1.07	147.97

All data obtained for four Coriell samples representing CN=1, 2, 3, 4, and 6. DNA (ng) denotes the total amount of input DNA used in the *CYP2D6* triplex CNV reaction. Two µL of each serial DNA dilution were used in each reaction. Each column includes the *CYP2D6* target region and the corresponding fluorescent dye. *TERT* served as the 2-copy reference assay. Calculated CNs shaded in green indicate values within the valid threshold (within ±0.25 of the expected integer value).

1.4 Supplementary Table 3

Reference Gene Assay	1022C (REF)	1022T (VAR)	4181C (VAR)
RNaseP	0.90	0.94	1.97
TERT	0.95	0.96	1.94

Calculated CN values for HG03313 (*CYP2D6*29/*154*) when tested with CN_1022 with RNaseP and *TERT* as the 2-copy gene reference assay. REF and VAR denote reference and variant, respectively.

2 Supplementary Figures

2.1 Supplemental Figure 1

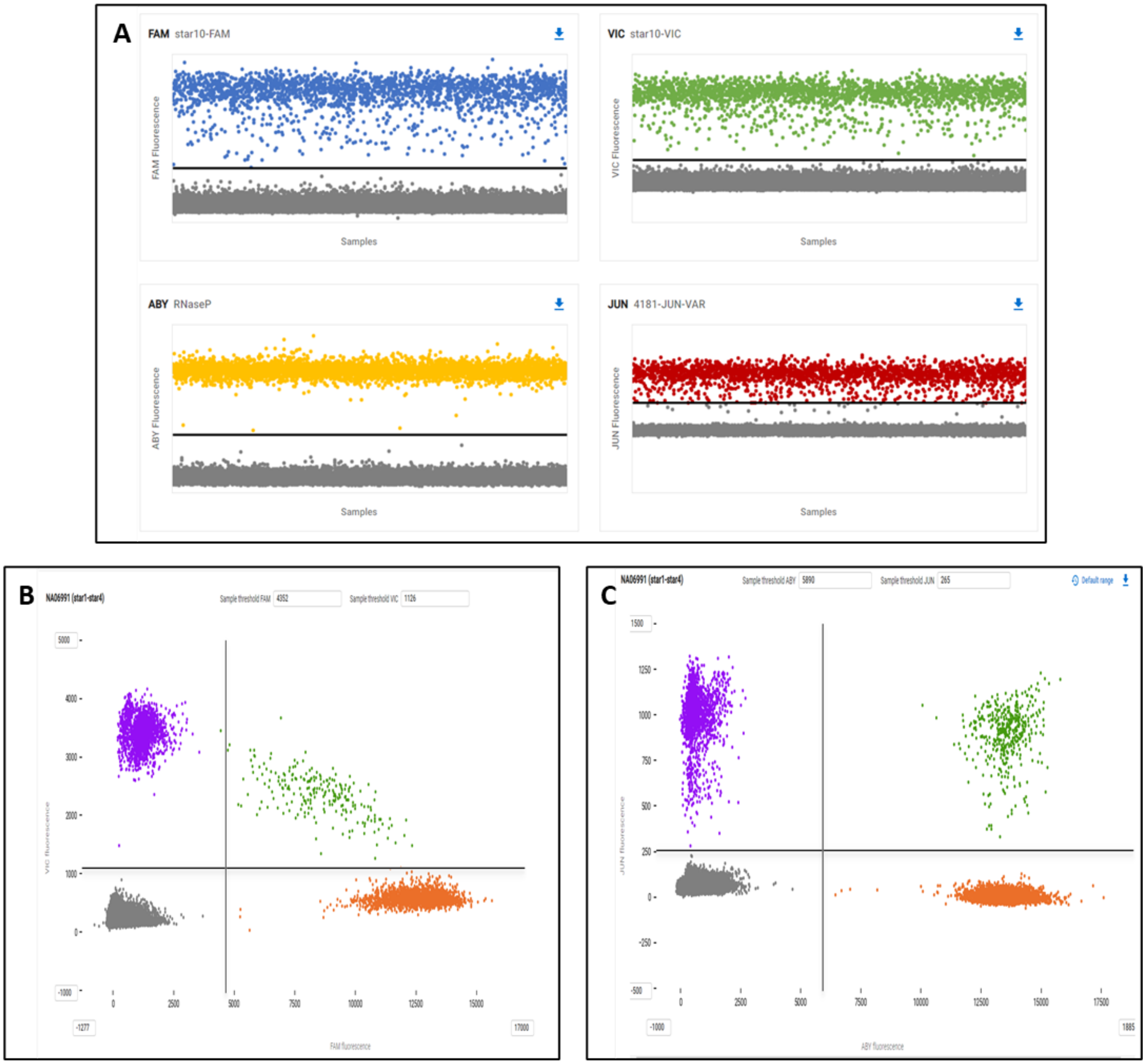

Coriell sample NA06991 (*CYP2D6*1/*4)* was tested with CN_100 as part of a series of assay validation experiments. Panel (**A**) shows the 1D plot with each florescent dye channel (FAM, blue; VIC, green; ABY, yellow and JUN, red). There are distinct clusters of positive (color) and negative (grey) reactions for each channel. Panel (**B)** and (**C)** represent 2D plots for FAM/VIC and JUN/ABY, respectively. Grey reactions are negative for both dyes, green are positive for both, purple is positive for one dye (VIC or JUN), and orange is positive for the other dye (FAM or ABY). There is distinct cluster separation for the 2D plots. The "arc" shape of the green cluster in **(B)** is consistent for genotyping assays because it represents double positive reactions with different allelic ratios present in each microreaction. As the reaction goes to endpoint, there is competition between the hybridization of the FAM and VIC probes (partition specific competition) (Whale et al., 2016).

2.2 Supplemental Figure 2

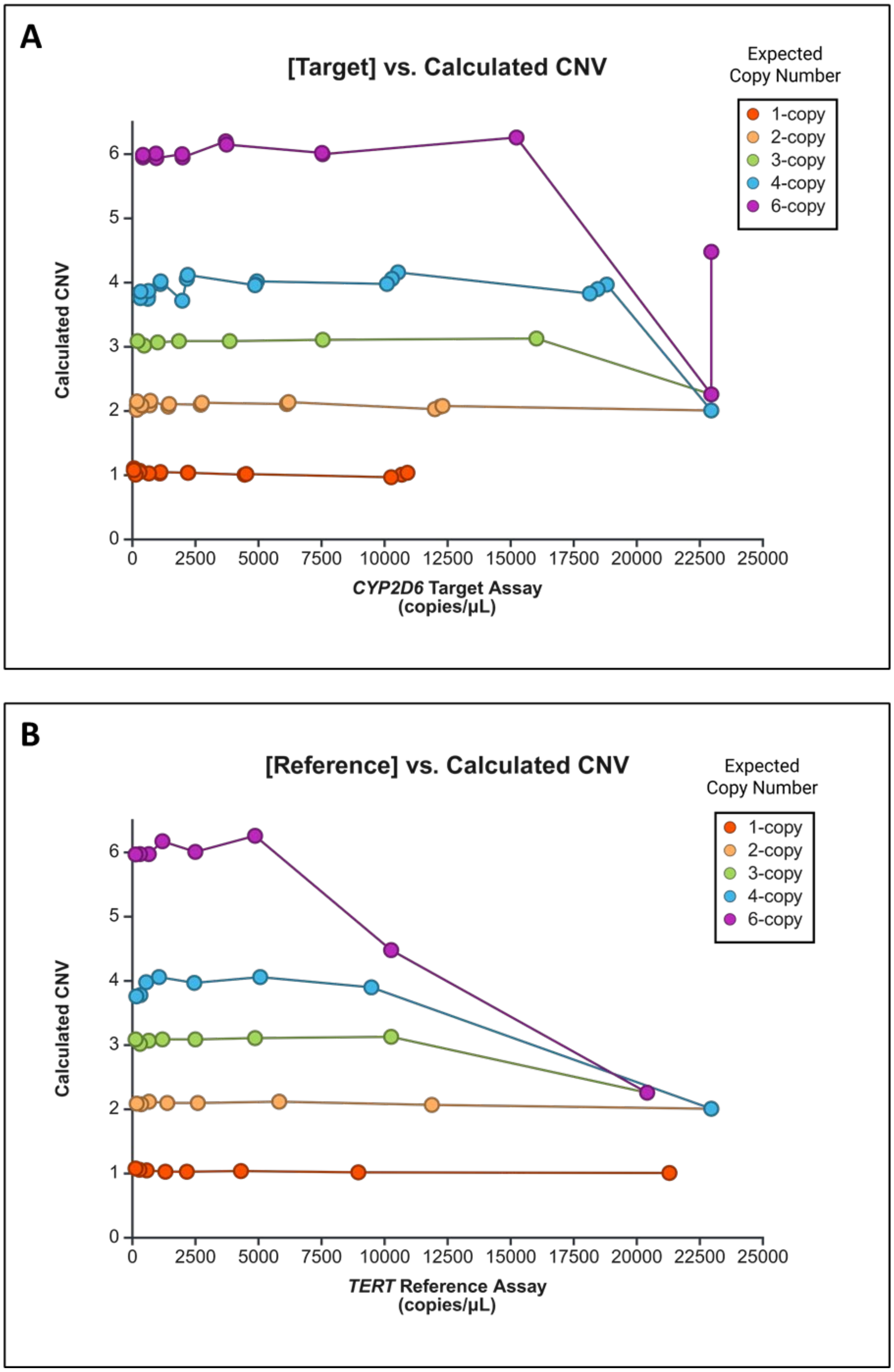

Graphical representation of the relationship between copies/μL of either the target assay (**A**) or the reference assay (**B**) and calculated CN. *CYP2D6* target regions included 5'UTR, intron 6, and exon 9, and *TERT* as the 2-copy reference gene assay.

2.3 Supplemental Figure 3

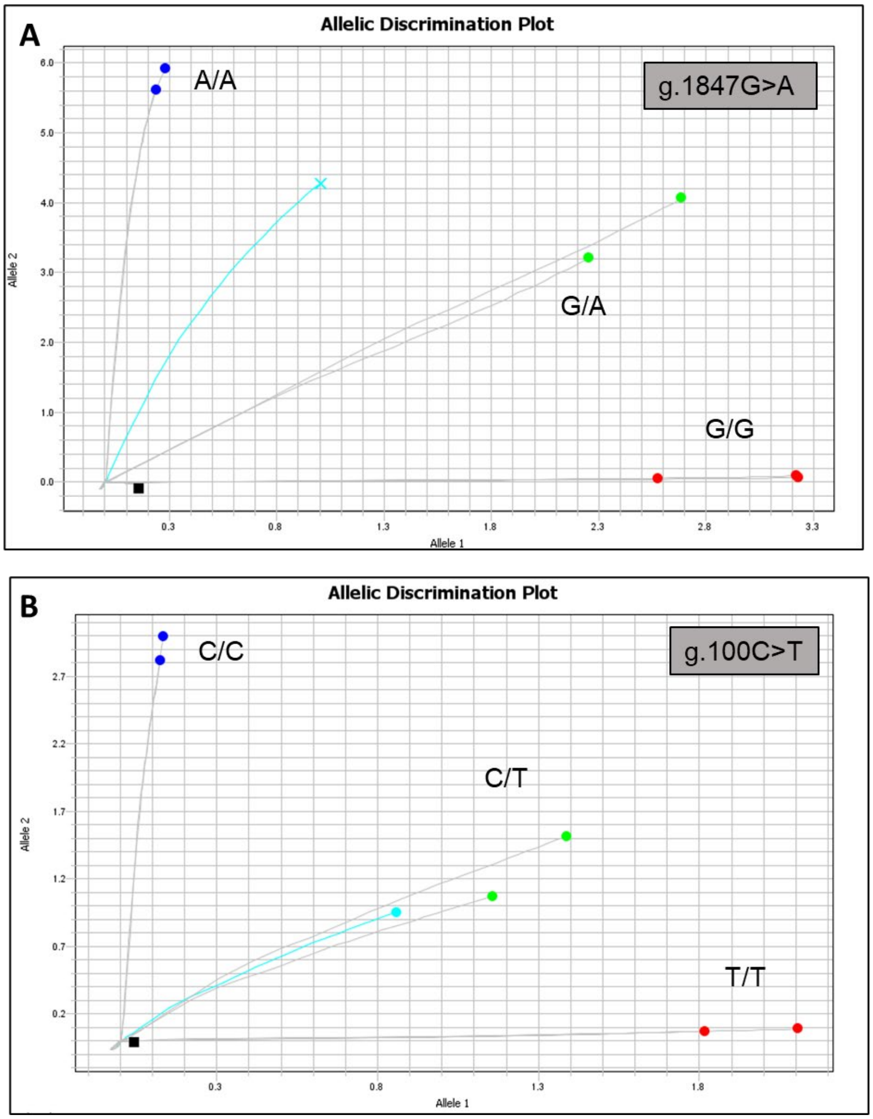

TaqMan™ allelic discrimination plots for HG00123 (*CYP2D6*1.005/*4.004)* which is highlighted by the cyan trace. The sample was genotyped for g.1847G>A, the *CYP2D6*4* core variant **(A)** and g.100C>T (**B**); this variant is present on most **4* alleles but is also part of several other star alleles including **10*. As shown in **(A)**, the amplification trace distinctly shifted towards the A/A cluster, rather than cluster with the G/A cluster, where it would be expected. In contrast, as shown in (**B**), the same sample clustered with other heterozygous samples for g.100C>T.
